# Astrocytes and neurons communicate via a monocarboxylic acid shuttle

**DOI:** 10.3934/Neuroscience.2020007

**Published:** 2020-04-20

**Authors:** Dirk Roosterman, Graeme S. Cottrell

**Affiliations:** 1 Ruhr Universität Bochum, LWL-Hospital of Psychiatry, Bochum, Germany; 2 School of Pharmacy, University of Reading, Reading, RG6 6AP, UK

**Keywords:** astrocyte neuron glucose transit, astrocyte neuron lactate shuttle, enzyme complexes

## Abstract

Since formulation of the Astrocyte-Neuron Lactate Shuttle (ANLS) hypothesis in 1994, the hypothesis has provoked criticism and debate. Our review does not criticise, but rather integrates experimental data characterizing proton-linked monocarboxylate transporters (MCTs) into the ANLS. MCTs have wide substrate specificity and are discussed to be in protein complex with a proton donor (PD). We particularly focus on the proton-driven transfer of l-lactic acid (l-lacH) and pyruvic acid (pyrH), were PDs link MCTs to a flow of energy. The precise nature of the PD predicts the activity and catalytic direction of MCTs. By doing so, we postulate that the MCT4·phosphoglycerate kinase complex exports and at the same time in the same astrocyte, MCT1·carbonic anhydrase II complex imports monocarboxylic acids. Similarly, neuronal MCT2 preferentially imports pyrH. The repertoire of MCTs in astrocytes and neurons allows them to communicate via monocarboxylic acids. A change in imported pyrH/l-lacH ratio in favour of l-lacH encodes signals stabilizing the transit of glucose from astrocytes to neurons. The presented astrocyte neuron communication hypothesis has the potential to unite the community by suggesting that the exchange of monocarboxylic acids paves the path of glucose provision.

## 1. Introduction

“The important point here is not so much to decide, based on the actual pieces of evidence, whether an hypothesis is right or wrong but rather to point out what is heuristically valid in it, what have we learned, what remains to be assessed, what new hypothesis can be proposed and which experiments are critical for it” [1]. The rationales leading to the Astrocyte-Neuron Lactate Shuttle (ANLS) hypothesis and the Neuron-Astrocyte Lactate Shuttle (NALS) were deduced from the common understanding of glucose metabolism [2],[3]. Textbooks didactically sort glycolytic enzymes by the gradual degradation of the carbon backbone. Free diffusion of substrates, products and enzymes is the underlying rational connecting glycolytic enzymes. However, glycolytic enzymes are organized in complexes and compartments. Moreover, a concept based on free diffusion, has to collapse by extrapolating the line of enzymes to membrane-anchored enzymes, such as proton-linked monocarboxylate transporters (MCTs), as membranes block free diffusion.

Experimental characterization of MCTs, under the premise of free diffusion would require that the MCTs be removed from the membrane before investigating. Doing so, MCTs would then be characterized as enzymes reversibly catalysing the equilibrium between monocarboxylic acid (R-COOH) and hydrated monocarboxylic acid (R-COO^−^ + H^+^[H_2_O]_n_). MCTs would be one of the fastest enzymes sorted and would have to be re-classified into the family of carbonic anhydrases (CA).

Our concept of Astrocyte Neuron Communication (ANC) is guided by the tentative 4^th^ law of thermodynamics. This law of nature predicts that a flow of energy is sufficient to form ordered structures [4]–[6]. Enzyme complexes are examples of highly ordered structures. Acids, such as carbonic acid (H_2_CO_3_), carry an active proton (H^+^). An active proton is an energy entity, which immediately reacts with water or is transferred to a coupled enzymatic reaction. An intra-complex transfer of H^+^ saves and transfers the hydration energy to the coupled enzymatic reaction [7]. Thus, whereas ANLS and NALS are based on concepts whereby enzymes catalyse a process leading to maximal entropy and transporters to a concentration equilibrium, ANC uses proton-linked MCTs directly coupled to glucose metabolism.

It is well known that energy in the form of ATP, provided by glucose metabolism, is consumed by Na^+^/K^+^-ATPases to create a Na^+^ gradient. The Na^+^/glucose symporter uses this Na^+^ gradient to re-import glucose from pre-urine. In turn, the Na^+^/glucose symporter catalyzed reaction creates a glucose gradient or negative entropy [8],[9]. In ANC, energy in form of H^+^ is provided by nascent acids, such as carbonic acid. As will be discussed later, MCT1 and carbonic anhydrase II (CAII) are functionally linked and are best understood as a coupled monocarboxylic acid/carbonic acid antiporter. The permanent provision of carbonic acid acts as an energy flow, enabling MCT1 to create negative entropy. Similar to Na^+^/glucose symporter, the biological function of MCTs best understood in context of an organism and not as single enzyme. ANC is biophysical concept, where glucose metabolism is the reverberatory activity inducing cell assembly and a flow of energy inducing ordered structures [4],[10],[11].

Before considering ANC, we must first explore the properties of MCTs present on astrocytes and neurons.

## 2. Astrocytic and neuronal MCTs

In muscle, heart and cancer cells, MCT1 is an importing transporter [12]–[14] and in pancreatic β-cells MCT1 catalyzes import of pyruvic acid (pyrH), triggering insulin secretion [15]. MCT1 contains a CAII-binding domain. CAII acts as “proton-antenna” accelerating MCT1 catalyzed import of R-COOH [16]–[18]. In contrast to ANLS, ANC considers MCT1 in complex with CAII. The formation of the complex predicts that MCT1 unidirectionally imports R-COOH depending on astrocytic H_2_CO_3_ flow.

In glycolytic cells, MCT4 is an exporting transporter [19]. We have previously discussed that phosphoglycerate kinase (PGK) catalyzes a nucleophile substitution [7]. The mechanism of nucleophile substitution predicts that the intermediate product of PGK catalyzed reaction is the proton carrier molecule 3-phosphoglyceric acid. Additionally, we claimed that MCT4·PGK unidirectionally exports R-COOH depending on glycolysis rate [7].

Thus, we consider that MCT1·CAII imports R-COOH and MCT4·PGK exports R-COOH, at the same time in the same astrocyte. An identical charaterization of MCT1 and MCT4 activity was recently published by Lynch et al. [20].

Located at presynaptic and postsynaptic sites in mice, MCT2 is a major neuronal MCT [21]. CAIV is a cell surface glycosylphosphatidylinositol (GPI)-anchored protein and is the best proton donor (PD) candidate for MCT2 [22],[23]. GPI anchored proteins are enriched in cholesterol rich microdomains which quench carbon dioxide membrane diffusion [24]. Thus, we postulate that the activity of MCT2·CAIV complexes is not directly coupled to neuronal carbonic acid flow.

MCT2 is also expressed in kidney and liver; here an interesting PD candidate is the microdomain-located Na^+^/K^+^-ATPase [25], as the exported Na^+^ is a Lewis acid participating in the H_2_CO_3_/HCO_3_^−^ + H^+^[H_2_O]_n_ equilibrium. Due to an as yet undefined mechanism, ANC sets the activity of MCT2 depending on neuronal activity. MCT2 is the so called “pyruvate transporter”. Protonated MCT2 demonstrates ten-time higher affinity to pyruvate (pyr^−^) over l-lactate (l-lac^−^) [26],[27]. Based on the substrate specificity of MCT2, ANC discusses the transfer of l-lacH and pyrH.

The massive increase in cytosolic l-lac^−^ during astrocytic glycogenolysis is one of the rationales for the ANLS hypothesis. By incorporating astrocytic MCT1·CAII complexes as importing and the MCT4·PGK complexes as exporting, allows the concepts of a feedback mechanism and cell-to-cell communication via monocarboxylic acids. Thus, the mechanism of ANC also comprises neuron to astrocyte monocarboxylic acid transfer and the NALS hypothesis. In ANC, MCT2 the major neuronal transporter of the adult mice brain, is functionally connected to CAIV and acts as importing transporter. ANC predicts that MCT2·CAIV is formed to import monocarboxylic acids, but ANC does not exclude functional connection of MCT2 to other PDs, which can change the catalytic direction of MCT2 from import to export. Moreover, MCT expression is species specific and changes during brain development. Thus, as neurons possess the ability to form a range of different MCT complexes, it stands to reason that some of these complexes must mediate monocarboxylic acid transfer from neurons to astrocytes.

## 3. Encoding the signal

Brilliant data from animal models of aversive training and fear conditioning have demonstrated that astrocytic glycogenolysis is essential for memory formation and consolidation (reviewed in [28],[29]). Furthermore, pharmacological inhibition of glycogenolysis blocks the formation of work memory [30] and genetic knockdown of MCT1 or MCT4 can be overcome by l-lac^−^ injection [31]. Thus, different groups using different animal models consolidate l-lac^−^ as the essential component in memory formation and consolidation.

Characterization of the kinetics of MCTs revealed a three-step reaction. First, the energy of a proton is transferred. Second monocarboxylate binds and third, MCTs catalyze the charge-neutral membrane transfer of R-COOH [32],[33]. The characterization of the kinetics indicates that energy transfer (proton provision) is the pacemaker of monocarboxylic acid transfer whereas environmental monocarboxylate concentration is secondary. In other words, the amount transferred monocarboxylic acids primarily depends on neuronal activity, whereas astrocytic glycogenolysis is secondary. So, how can astrocytic glycogenolysis be essential for memory formation and consolidation?

Glycogenolysis greatly affects the cytosolic l-lac^−^/pyr^−^ ratio in favour of l-lac^−^ [34]. In ANC, the signal triggering memory consolidation is encoded in the l-lac^−^/pyr^−^ ratio and not by the amount of imported monocarboxylic acids. The signal can be encoded by an increased presence of l-lac^−^ or the relative absence of pyr^−^. Although, we agree that detection of “an absence” of pyr^−^ may sound confusing, cells are permanently exchanging glucose and glucose metabolites and must therefore detect changes in both directions to adapt to environmental changes. Glucose, l-lacH and pyrH must be understood as distinct signalling molecules, triggering distinct signal cascades.

The primary l-lac^−^- and pyr^−^-detecting enzymes are the isoforms of lactate dehydrogenase (LDH). Muscle LDH (LDH-m) catalyzes the reduction of pyr^−^, whereas heart LDH (LDH-h) catalyzes the oxidation of l-lac^−^ [35]. Thus, the substrate specificity of the LDH isoforms already sterically separates the metabolism of the different monocarboxylic acids. Moreover, similar to MCT complexes, the LDH isoforms also form complexes, with for example glyceraldehyde-3-phosphate [36],[37]. In this way, the LDH complexes can channel energy metabolites. This mechanism was first formulated in the metabolite channelling hypothesis [38]. We used this hypothesis to develop the proton transport chain hypothesis, which focuses on NADH-H^+^ as a carrier of the energy entity H^+^ [7].

The proton transport chain hypothesis was based on the characterization of the kinetics of MCTs [32],[33]. Thus, the provision of the active proton or the nascent acid is the initiating step of the coupled enzymatic reaction. On basis of this hypothesis, LDH-m is a proton acceptor (PA) and the reduction of pyr^−^ to l-lac^−^ primarily depends on the provision of NADH-H^+^ and LDH-h is a PD and the oxidation of l-lac^−^ to pyr^−^ provides the proton carrier enzyme NADH-H^+^. A suitable approach to determine the intra-complex H^+^ would be to use deuterium labelled substrates. However, the detection of the intra-complex proton in certain MCT·PD complexes would not be possible due to the nature of the H^+^. For example, in the MCT4·PGK complex, the H^+^ would be provided by the protons dissolvated in the substrate, 1,3-bisphosphoglycerate [7],[39] and thus, covalently labelling could not be achieved. However, if we considered that all MCTs used the same proton-driven mechanism, then the mitochondrial LDH-h·MCT1 complex in the inner membrane of mitochondria [40] could be used to test this hypothesis. Although challenging, lactate-oxidising mitochondria can be isolated and the intra-complex H^+^ could simply be “traced” using l-lac^−^ labelled at the alpha hydroxyl (-OH) with deuterium. The presence of deuterium in the mitochondrial LDH-h·MCT1 complex could then be confirmed. As, the “proton shuttle” of CAII has been shown as a requirement for the augmentation of MCT1 and MCT4 activity [16], it would be interesting to determine whether the silencing of PGK or a catalytically inactive PGK mutant (PGK-T378P) would affect the activity of MCT4.

**Figure 1.**
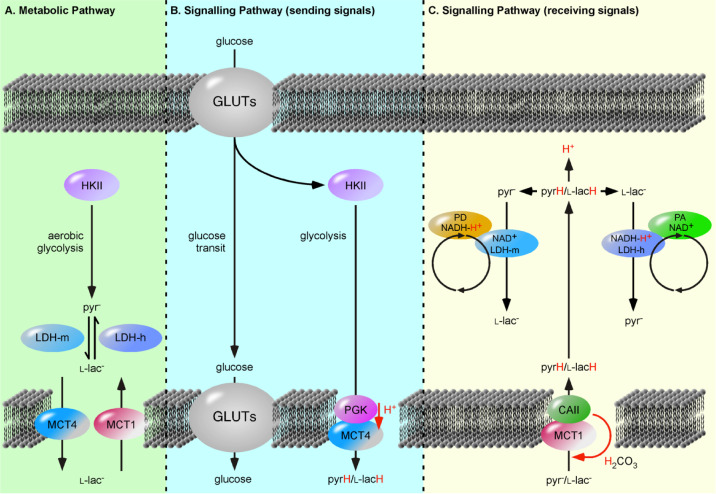
The Processing of Glucose as a Metabolic versus Signalling Pathways. (**A**) The metabolic pathway is understood as a solely cytosolic process, were hexokinase II (HKII) is set as first enzyme and initiates the gradual degradation of the carbon backbone of glucose to pyruvate (pyr^−^). The extrapolation of so-called “aerobic glycolysis” to include lactate dehydrogenases (LDHs) and proton-linked monocarboxylate transporters (MCTs) encounters two pitfalls. First, the reactions are illustrated with arrows indicating an equilibrium reaction catalyzed by single enzyme and not as two independent and sterically separated metabolic pathways. Second, following a sequential pathway, i.e., that the product of enzyme A is substrate of enzyme B, results in MCT catalyzed transfer of l-lactate (l-lac^−^), instead of monocarboxylic acids. (**B, C**) Glucose metabolism as signalling pathways can be divided in a “sending” pathway and a “receiving” pathway. The transferred signalling molecules are glucose, pyruvic acid (pyrH) and lactic acid (l-lacH). In the “sending” pathway, the MCT4·phosphoglycerate kinase (PGK) complex exports l-lacH and pyrH depending on the glycolysis rate or PGK activity. The “receiving” pathway utilizes the MCT1·carbonic anhydrase II (CAII) complex, which import monocarboxylic acids depending on cellular carbonic acid (H_2_CO_3_) flow. The imported l-lacH and pyrH are detected by heart lactate dehydrogenase (LDH-h) and muscle LDH (LDH-m) complexes, respectively. Here, LDH-m acts as a proton acceptor (PA) protein, whereas the LDH-h acts as proton donor (PD) protein and catalyzes the oxidation of l-lac^−^ forming the proton carrier, NADH-H^+^. The LDH-m complexes are considered part of the glycolytic pathway, detecting the metabolism of glucose. The separation of unidirectionally catalyzed reactions into distinct pathways provides an explanation for why pancreatic β-cells release insulin in response to imported pyrH, similar to glucose [15].

In ANC, LDH complexes are understood as metabolic signalling domains linking glucose metabolism to redox- and pH-sensitive signalling pathways [7]. One way to test this hypothesis and the ANC model would first be to analyse of the existence of the postulated PD·PA complexes. An indication of complex formation could be achieved using “proximity ligation assays” [41],[42]. Although not an indicator of direct interaction, this assay could be used to determine whether the two proteins are in close proximity (< 40 nm) and which proteins are located in proximity of the complex linking glucose metabolism to pH- and redox-sensitive signalling pathways. Considering glucose metabolism as a purely metabolic pathway prevents the understanding of glucose metabolism as signalling pathway (Figure 1) (reviewed in [43]. Pullen et al. demonstrated that import of pyrH, not l-lacH, triggers insulin release from pancreatic β-cells, implying that pyr^−^ is coupled to signalling cascades similar to glucose [15]. The relative absence of pyr^−^ is detected as “low glucose” and cells adapt by upregulation of glucose transporters (GLUTs) [44]. In addition, neuronal MCT2 demonstrates a ten times higher affinity for pyr^−^ over l-lac^−^ and thus, the primary role of MCT2 is to “clean” pyr^−^ from the environment, implying that pyr^−^ is the critical factor (Table 1).

**Table 1.** Affinity of MCT family members for l-lactate and pyruvate. K_M_ values (mmol/L) are from [13]. More recently*, K_M_ values for MCT4 (determined using the FRET sensors, Lactonic and Pyronic) characterized MCT4 as high affinity transporter [45].

Monocarboxylate	MCT1	MCT2	MCT4	MCT4*
l-lactate	3.5	0.74	28	1.7–0.7
Pyruvate	1.0	0.08	153	4.2

## 4. Astrocytes transit glucose

Tadi and co-worker analysed the expression and transcription of enzymes participating in ANLS in a rodent model of fear conditioning [44]. Neuronal and astrocytic GLUTs, the first enzymes of glucose metabolism, are upregulated during fear conditioning. Furthermore, siRNA knockdown of GLUT family members reduces glucose influx in HepG2, clearly indicating that GLUTs regulate glucose flow [46]. Nevertheless, hexokinase 2 (HK2) is well known to regulate glycolysis rate. So, how can GLUT expression levels play a major role in memory consolidation, when HK2 is the pacemaker?

Astrocytes are part of the blood brain barrier [47], cover the surface of cerebral blood vessels, have projections in perisynaptic areas of neurons and are the preferential site for glucose uptake from the blood [48]–[50]. The high coverage of capillaries indicates that glucose must pass though astrocytes to reach neurons. At least two ways of neuronal glucose supply are possible: (i) blood glucose is provided via the interstitial fluid (ISF) and diffuses into the three dimensional space or (ii) astrocytes act as glucose transit cells [51].

Our ANC hypothesis postulates that the exchange of R-COOH paves the path of glucose provision and astrocytes act as glucose transit cells. Our argument is supported by the rational that ISF is continuously exchanged with the cerebrospinal fluid (CSF) [52]. CSF has a glucose concentration of 1.8–2.9 mM [53]. Moreover, Gjedde et al. determined low glucose concentration of the ISF (2.6 ± 0.2 mM) [54]. The low glucose concentration actually excludes ISF as source of neuronal glucose supply.

**Figure 2.**
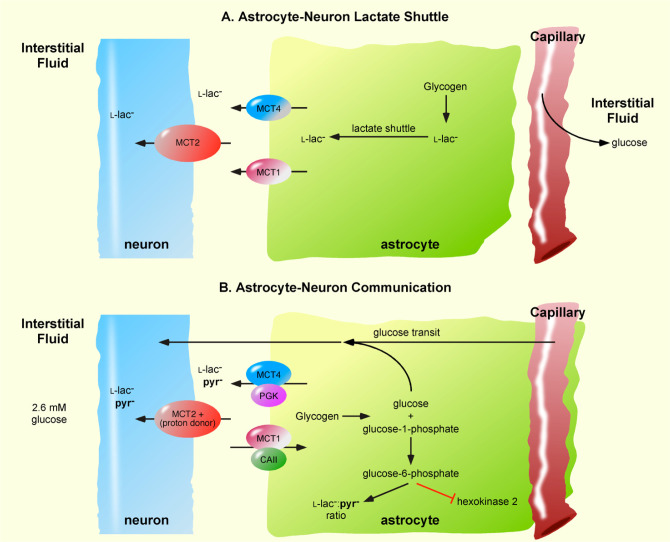
Comparison of the Astrocyte-Neuron Lactate Shuttle and Astrocyte-Neuron Communication Models. (**A**) In the Astrocyte-Neuron Lactate Shuttle model, astrocytic monocarboxylate transporter 1 (MCT1) and MCT4 as export the lactate (l-lac^−^) and neuronal MCT2 imports l-lac^−^. Glucose is provided to neurons via the diffusion of blood glucose into the interstitial fluid. Astrocytic glycogenolysis provides an end product l-lac^−^ for export to neurons. (**B**) Astrocyte-Neuron Communication uses proton-linked MCTs [7], with MCT1 in complex with carbonic anhydrase II (CAII) and MCT4 in complex with phosphoglycerate kinase (PGK). MCT1·CAII unidirectionally imports monocarboxylic acids (pyruvic acid and lactic acid) depending on the astrocytic carbonic acid flow, whereas MCT4·PGK exports monocarboxylic acids depending on PGK activity or the astrocytic glycolysis rate. The low glucose concentration in the interstitial fluid excludes neuronal glucose provision via this route. Instead, astrocytes transit blood glucose to neuronal compartments. Astrocytic glycogenolysis produces glucose and glucose-1-phosphate, the latter being converted to glucose-6-phosphate the activity of phosphoglucomutase. Glucose-6-phosphate inhibits hexokinase 2 activity and thereby blocks astrocytic metabolism of glucose and facilitates the transit of glucose to neurons.

Astrocytic glucose transit is also supported by the well known mechanism of glycogenolysis. Astrocytic glycogenolysis increases cytosolic l-lac^−^, but the first step is hydrolysis of glycogen to glucose-1-phosphate and glucose [55]. Glucose-1-phosphate is then converted to glucose-6-phosphate, which efficiently inhibits HK2 [56]. Thus, during glycogenolysis, glucose-6-phosphate blocks astrocytic metabolism of glucose and additional glucose is provided. This mechanism allows astrocyte to transit glucose to neurons at a concentration higher than blood glucose (Figure 2).

Taken together, ANC is an alternative hypothesis to the well-established ANLS hypothesis. In contrast to ANLS, ANC considers proton-linked MCTs to be in complexes with PDs. The nature of the PD predicts the activity and catalytic direction of associated MCT. The suggested direct provision of an acid/active proton to the active side of MCT pumps the hydration energy into the MCT catalyzed process. This theoretical mechanism enables MCTs to transfer R-COOH against a pH and monocarboxylate gradient. The ANLS and NALS hypotheses are based on reversibly acting transporters allowing MCTs either to import or to export depending on a concentration gradient or enzyme affinity, respectively. In contrast, ANC based on unidirectionally acting enzyme complexes, formed by a flow of energy. Thus MCT1·CAII, driven by the permanent flow of carbonic acid imports R-COOH and at the same time in the same astrocyte, MCT4·PGK, driven by PGK activity exports R-COOH. ANC discusses that at least pyrH and l-lacH have to be considered to be continuously exchanged. This simple but important difference between ANLS and ANC, could be tested by incubating astrocytes in medium containing only trace amounts of ^13^C-labelled pyr^−^ and ^2^H-labelled l-lac^−^ and analysing uptake. Whereas ANLS understood lactate to be food for hungry neurons, ANC understands l-lacH and pyrH as signalling molecules, paving the path for glucose provision [2]. Glucose metabolism is a good candidate to be the reverberatory activity tending to induce growth process and metabolic change stabilizing neuronal processes [10]. The relative absence of pyrH in neuronal and astrocytic imported R-COOH was suggested as critical signal for cell assembly.

## 5. Discussion and conclusion

During the development of our theory of ANC and its role in memory formation and consolidation, we may have omitted mentioning many brilliant and highly informative manuscripts in this area. However, our impact in this scientific field is turning the catalytic direction of MCT1 from export to import, which changes the ANLS hypothesis from “food for hungry neurons” to astrocyte neuron communication, ANC.

ANC hypothesis is in full agreement with the data supporting ANLS, but simply provides alternative interpretation. First, by splitting R-COOH membrane transfer into distinct exporting and importing complexes, the metabolism of glucose to l-lacH is no longer the opposite reaction of importing l-lacH and gluconeogenesis, but two distinct metabolic processes. In contrast to ANLS, which is a concept based on free diffusion and merges export and import of l-lacH at reversibly acting MCTs producing a deadlock situation, ANC allows activated neurons to consume more glucose and more l-lacH, at the same time in the same cell. Suzuki et al. investigated the genetic knockdown of proton-linked MCT1, MCT2 or MCT4 on long-term memory formation [31]. They found that the genetic knockdown of MCT1 and MCT4 is rescued by lactate injection. However, the brilliant data were interpreted on the basis that both MCT1 and MCT4 act as exporting transporters. If this was true, the knockdown of one exporting transporter should be compensated by the other one.

We assume that similar mechanisms of R-COOH signalling take place in astrocytes and neurons. In line with ANLS, genetic knockdown of MCT4 blocks R-COOH export. In contrast to ANLS, we place neuronal MCT2 and astrocytic MCT1·CAII as importing transporters. Thus, an injection of lactate greatly changes the l-lac^−^/pyr^−^ ratio in favour of l-lac^−^ and then astrocytic MCT1 and neuronal MCT2 mediate the detection of the relative absence of pyr^−^. ANC provides an avenue whereby MCT1·CAII triggers a feedback mechanism. Following this path, MCT1·CAII activity primarily impacts astrocytic signalling cascades that trigger memory formation and consolidation, such as improved glucose transit. Suzuki et al. demonstrated that the knockdown of MCT1 is rescued by injection of high glucose (1 day after training) [31]. In ANC, high glucose increases glucose flow and mimics upregulation of astrocytic GLUTs. Thus, ANC provides a theoretical mechanism to explain this observation that cannot be explained by ANLS.

MCT1 knockdown can also by rescued by lactate injection [31]. There are a number of possible explanations for this observation. Firstly, in the study the knockdown on MCT1 was only approximately 50%, an injection of lactate would greatly increase the concentration and perhaps be sufficient to rescue the impact of knockdown on MCT1 function. Alternatively CAII, now freed from the MCT1·CAII complex could associate with MCT4 reversing the catalytic direction of MCT4 from export to import. We strongly believe that our concept of astrocyte glucose transit opens avenues to integrate data on glial cell signalling and intercellular communication [57].
